# NLRC5 Functions beyond MHC I Regulation—What Do We Know So Far?

**DOI:** 10.3389/fimmu.2017.00150

**Published:** 2017-02-17

**Authors:** Szilvia Benkő, Elek Gergő Kovács, Felix Hezel, Thomas A. Kufer

**Affiliations:** ^1^Faculty of Medicine, Department of Physiology, University of Debrecen, Debrecen, Hungary; ^2^Institute of Nutritional Medicine, University of Hohenheim, Stuttgart, Germany

**Keywords:** innate immunity, MHC, NLR, IFN, NF-κB, inflammasome

## Abstract

NLRC5 is a member of the NLR family that acts as a transcriptional activator of MHC class I genes. In line with the function of several related NLR proteins in innate immune responses, there is, however, also ample evidence that NLRC5 contributes to innate and adaptive immune responses beyond the regulation of MHC class I genes. In human and murine cells, for example, NLRC5 was proposed to contribute to inflammatory and type I interferon responses. The role of NLRC5 in these and other cellular processes is hitherto still not well understood and blurred by discrepancies in the reported data. Here, we provide a detailed and critical discussion of the available experimental data on the emerging biological functions of NLRC5 in innate immune responses in men and mice. Better awareness of the multiple roles of NLRC5 will help to define its overall contribution to immune responses and cancer.

## Introduction

In the past decades, we witnessed a rapid increase in our knowledge of the molecular details of innate and adaptive immune signaling pathways, including the identification of the cognate innate immune receptors, their signaling proteins, and the main effector responses of immunity. Several groups of pattern-recognition receptors (PRRs) were identified, such as the cytosolic nucleotide-binding domain (NBD), leucine-rich containing proteins (NLR), which are evolutionary conserved proteins that serve as sentinels for microbes and cellular stress to trigger induction of innate and adaptive immunity. NLRs are tripartite domain-containing proteins that emerged as important sensors for pathogen-derived microbe-associated molecular patterns (MAMP) and endogenous danger signals [danger-associated molecular patterns (DAMPs)] (1). The archetypical domain organization of an NLR protein consists of an N-terminal death-fold domain [mostly a pyrin domain or a caspase activation and recruitment domain (CARD)], a central ATPase domain of the signal transduction ATPases with numerous domains clade {assigned as NACHT domain [from NLP family apoptosis inhibitor protein, class II transcriptional activator (CIITA), HET-E, and TEP1]}, and C-terminal leucine-rich repeats (LRR) of various lengths. Notably, mammalian NLRs are close relatives of immune proteins found in plants that act as sensors for pathogen-expressed effector proteins (2). In the human genome, at least 22 NLRs and in the mouse genome 34 such proteins are encoded, and many of these still are poorly characterized. According to our view, the mammalian NLR proteins can be subdivided in three functional classes: (I) NLRs that convert microbial sensing to transcriptional reprograming in the host cell, resulting in pro-inflammatory responses and expression of bactericidal activities. This is exemplified by NOD1 and NOD2 that are prototypic PRRs (3). (II) NLRs that induce formation of a multiprotein complex which was named “inflammasome” that in turn recruits and activates the zymogen pro-caspase-1, resulting in caspase-1-mediated processing of the cytokines IL-1β and IL-18 and the induction of pyroptotic cell death (examples being NLRP3 and NLRP1) (4, 5). (III) NLRs that can translocate into the nucleus and act as transcriptional transactivators. The most prominent member of this latter family is the CIITA. This protein is essential to drive transcription of major histocompatibility complex class II (MHC II) genes by acting as a transactivator on MHC II promoter region where it binds to indirectly, by interaction with an accessory DNA-binding protein complex, the MHC enhanceosome (6). Recently, the NLR protein NLRC5 was shown to have a similar function in the regulation of MHC class I genes (7–11). Taken together, this nicely exemplifies that NLRs contribute to diverse biological functions (12). Here, we focus on the NLRC5 protein and our current understanding of its biological functions.

NLRC5 was first cloned and characterized in 2010, independently by five groups (7, 13–16). It is meanwhile well established that NLRC5 shuttles to the nucleus where it can induce the transcription of MHC class I genes, in most, albeit not all, cell types (17–19). MHC molecules present peptide antigens to T cells of the adaptive immune repertoire that interact with MHC–peptide complexes with their cognate T cell receptors. MHC class I molecules thereby present cytosolic-derived peptides to CD8^+^ cytotoxic T lymphocytes (CTL), whereas MHC II molecules present vesicular-derived peptides to CD4^+^ T helper cells. NLRC5 seems to act in a similar manner as CIITA, and its activity is regulated mainly at the transcriptional level. Indeed, it was shown that expression levels of NLRC5 and MHC class I genes correlate in mouse and human cell lines and tissue (7, 9, 10). Many cancer entities show reduced or absent MHC I expression, and this is discussed as an important event to circumvent CTL-mediated antitumor immune responses. As NLRC5 regulates MHC class I expression, it might be relevant in the malignant transformation process and could eventually be used as intervention to enhance antitumor immunity (20–22). Indeed, induced expression of NLRC5 was recently correlated with enhanced immunogenicity and reduced tumor progression in a mouse model of melanoma (23).

**Figure 1:**
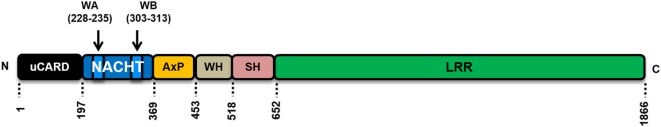
**Schematic representation of the human NLRC5 protein structure and domain organization**. uCARD, untypical caspase activation and recruitment domain (CARD); this domain shows only low sequence similarities to canonical CARD domains. A nuclear localization signal is located in this region. NACHT domain, involved in the nucleotide bindings and presumable important for oligomerization and activation of NLRC5. WA and WB are Walker A and Walker B motifs, respectively. The WA motif is responsible for nucleotide triphosphate binding; WB motif is responsible for nucleotide triphosphate hydrolysis. WH, winged helix domain. SH, superhelical domain, contains eight α-helices, function unknown. LRR, leucine-rich repeat, protein–protein interaction domain, responsible for ligand binding in other pattern-recognition receptors.

Interestingly, although NLRC5 shares sequence homology to CIITA, it possesses a rather atypical N-terminal domain, which is completely distinct from the CIITA N-terminal domain and that of other NLRs. Furthermore, NLRC5 has the longest LRR domain of all human NLR proteins (16, 24, 25) (Figure 1). This might suggest that NLRC5 has further functions in human cells, and indeed, most of the initial reports on NLRC5 did not reveal its important function in antigen presentation, but rather suggested that NLRC5 contributes to cell-autonomous immune responses (13–16).

**Figure 2:**
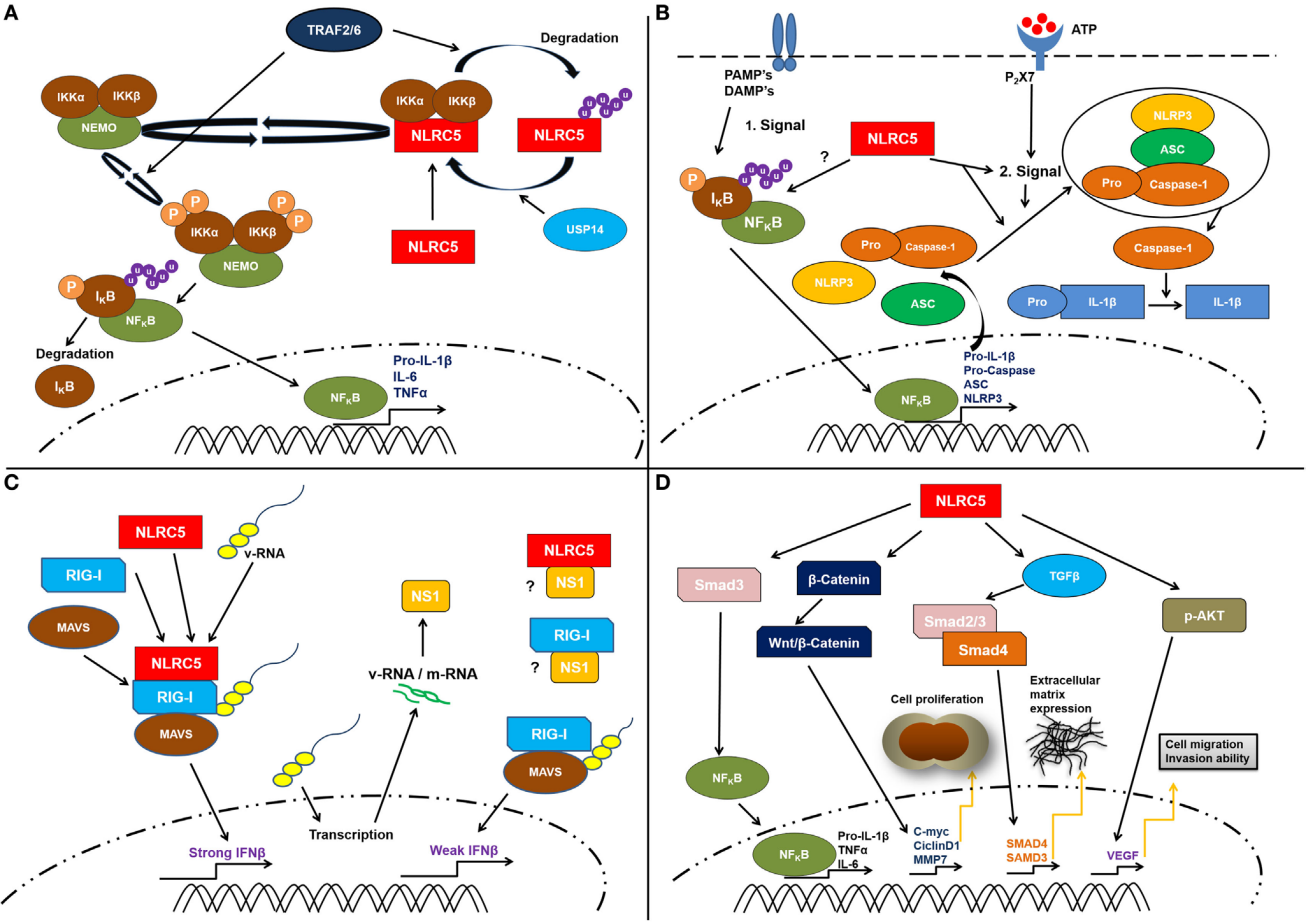
**Schematic representation of the proposed cellular functions of cellular NLRC5**. **(A)** Several studies suggest a contribution of NLRC5 to the nuclear factor kappa B (NF-κB) pathway. This might involve specific ubiquitination of NLRC5. A direct interaction between the IKKα/β and NLRC5 resulting in IKKα/β, which is unable to bind to NEMO (IKKγ) as well as reduced autophosphorylation and kinase activity was reported. Following lipopolysaccharide treatment, the TNF receptor-associated factors 2/6 (TRAF2/6) mediate NLRC5 ubiquitination. This leads to the degradation of NLRC5 that consequently frees IKKα/β complex and activates NF-κB pathway. **(B)** Possible role of NLRC5 in controlling inflammasome activation. The molecular mechanisms how this is achieved remains elusive. NLRC5 might affect priming by the regulation of NF-κB responses or might directly interfere with inflammasome activation. However, available data suggest that NLRC5 is more likely involved in the complex forming step of the inflammasome activation. **(C)** In antiviral responses, some studies suggest that NLRC5 interacts with retinoic acid-inducible gene I (RIG-I) and MAVS and might be able to enhance type I interferon responses. However, also contradicting data, suggesting a role of NLRC5 I inhibiting type I interferon responses has been presented. Several studies showed that in the absence of NLRC5, the IFNβ response is significantly weaker compared to the level observed when NLRC5 interacts with RIG-I and MAVS. **(D)** Finally, recent works suggest that NLRC5 can be linked to tumorigeneses *via* multiple pathways, such as β-catenin, TGF-β, and Akt, thereby resulting in changed cell proliferation and extracellular matrix deposition. However, these functions await validation at present. It should be mentioned that most of the depicted functions await further clarification (see main text for discussion).

Here, we provide a detailed discussion of the available data on the functions of NLRC5 with a strong focus on its roles beyond the well-established and accepted function in regulating MHC class I gene expression (Figure 2).

## NLRC5 Functions Beyond MHC Regulation

Although there is significant progress in understanding the structure, the expression, and the transactivating function of NLRC5 in the context of MHC class I gene expression, reports about cytoplasmic functions and possible roles of NLRC5 in innate immune responses remain somewhat controversial and unresolved. Innate immune responses, including inflammatory cytokine production, are triggered by MAMPs or DAMPs and mainly are mediated *via* pro-inflammatory signal transduction pathways, inflammasome activation, or type I interferon responses. In the following sections, we discuss the reported functions of NLRC5 in these pathways.

## Pro-Inflammatory Responses

Since the discovery of NLRC5, there are multiple reports on functions of NLRC5 in the regulation of signal transduction pathways and pro-inflammatory cytokine production. Based on some early reports from 2010, describing that NLRC5 can inhibit nuclear factor kappa B (NF-κB) signaling (13, 14), the view of NLRC5 as a negative regulator of inflammatory responses has been established. Using reporter assays, it was shown that NLRC5 decreased NF-κB, interferon-sensitive response element (ISRE), and activator protein 1 signaling, and silencing of NLRC5 in the murine macrophage cell line RAW264.7 resulted in increased IL-6, TNF, RANTES (CXCL5), and IL-1β secretion (13, 14) and at the same time decreased secretion of the anti-inflammatory cytokine IL-10 (13). This inhibitory role of NLRC5 was explained by direct interaction between NLRC5 and IκB kinase alpha/beta (IKKα/β) that prevents the binding of NEMO (IKKγ) to the IKK subunits and inhibits their autophosphorylation and kinase activity (14). More recently, the same authors modeled NF-κB regulation by NLRC5 and revealed that reversible ubiquitination of NLRC5 has an important regulatory role in the activation of NF-κB signaling (26). They showed that following lipopolysaccharide (LPS) treatment, the E3 ligases TNF receptor-associated factors 2/6 (TRAF2/6) mediate NLRC5 ubiquitination on Lys1178. This K63-linked ubiquitination leads to the degradation of NLRC5 that frees IKKα/β complex and activates NF-κB pathway (26).

To determine the *in vivo* function of NLRC5, Kumar et al. generated NLRC5-deficient C57BL/6 mice by replacing exon 4 of *Nlrc5* with a neomycin-resistance gene cassette (27). However, bone marrow-derived dendritic cells (BMDCs) generated from wild-type and their *Nlrc5^−/−^* mice showed no differences in IL-6 and TNF pro-inflammatory cytokine production in response to LPS treatment or *Listeria monocytogenes* infection, questioning a physiological role of NLRC5 in pro-inflammatory responses in this cell type (27). Yao et al. generated another *Nlrc5^−/−^* mouse line in the C57BL/6 background, replacing exons 1–4 to a neomycin-resistant gene cassette (11). Consistent with the results from Kumar et al., they found that the expression of NF-κB-dependent genes did not change in BMDMs of NLRC5-deficient mice upon LPS treatment or *L. monocytogenes* infection (11). Similar results were obtained by Robbins et al. using C57BL/6 mice deficient in exons 3 and 4 of *Nlrc5* that encode a portion of the N-terminal domain and the entire NBD of NLRC5. They reported that, following LPS activation, cytokine levels of IL-6, TNF, and IL-1β were generally similar between WT and *Nlrc5^−/−^* BMDMs and peritoneal macrophages (28).

In 2012, Tong et al. generated a different NLRC5-deficient mouse line by deleting exon 8 of NLRC5 (29). This exon codes the functional domain of NLRC5 that inhibits NF-κB signaling (14). In agreement with the Kumar group, they found no differences in IL-6, TNFα, and IL-12 secretion between *Nlrc5^−/−^* and WT BMDCs stimulated with LPS (29). By contrast, when using embryonic fibroblasts (MEFs) derived from their *Nlrc5^−/−^* mice, they found enhanced IKK phosphorylation and NF-κB activation, supporting a negative effect of NLRC5 on the IKK pathway. Furthermore, they measured elevated mRNA levels of IL-6, TNF, and IL-1β in MEFs and peritoneal macrophages of *Nlrc5^−/−^* mice upon LPS activation (29). Interestingly, they found that NLRC5 deletion enhanced IL-6 expression in both MEFs and macrophages, but increased production of TNF was only observed in NLRC5-deficient MEFs but not in macrophages (29). Unfortunately, from the work, it is unclear what mouse background was used, impairing further interpretation of these results (29). An inhibitory role of NLRC5 on inflammatory cytokine production is further supported by work from Li et al. who demonstrated that silencing of NLRC5 in murine RAW264.7 macrophages results in increased IL-6 and TNF secretion after LPS activation (30).

Possible explanations for the different reported roles of NLRC5 in peritoneal macrophages, BMDMs, BMDCs, and MEFs might be the differential expression levels of NLRC5 in these cell types, the different availability of accessory proteins for NLRC5 function, and/or the different regulation of cytokine expression in these cells, as exemplified by TNF expression (31). A recent paper suggests that ubiquitination of NLRC5, which is different in these cell types such as murine peritoneal macrophages, BMMs, and BMDCs (26) may explain at least in part these discrepancies.

Taken together, NLRC5 is unlikely a pivotal regulator of pro-inflammatory responses under physiological conditions; however, available data strongly suggest that NLRC5 can affect the NF-κB pathway, in certain cell types. Interestingly, this function of NLRC5 might even be evolutionary conserved as a mutation in the promoter region of the NLRC5 homolog in chicken was associated with changes in NF-κB responses toward *Salmonella* infection (32) (Figure 2A).

Further research is needed to address and validate independently these points and to establish a function of NLRC5 in animal models or provide indications for correlation with human diseases.

## Inflammasome Activation

IL-1β and IL-18 are key inflammatory cytokines. These are produced in the cell as zymogens that are processed by caspase-1. The activation of caspase-1 is mediated by a fibril complex of the adaptor protein ASC that is nucleated upon homomeric complex formation of NLRP proteins, most importantly NLRP3. These NLRs are activated by multiple MAMPs and DAMPs in a still not understood manner involving changes in ion flux and redox potential [reviewed in Ref. (4, 5)].

As some of the above discussed studies also reported changes in IL-1β secretion, it is tempting to speculate that NLRC5 affects the function of inflammasomes or might be able to form an NLRC5-containing inflammasome.

While Tong et al. and Kumar et al. did not observe any differences in IL-1β secretion between *Nlrc5^−/−^* and WT peritoneal macrophages (27, 29), others have shown that NLRC5 contributes to NLRP3 inflammasome activation and inflammasome-dependent IL-1β secretion *in vitro* in response to a variety of NLRP3 stimulators. Short hairpin RNA (shRNA) and small-interfering RNA (siRNA) duplexes used to silence NLRC5 in human myeloid THP-1 cells and human primary monocytes significantly reduced the secretion of IL-1β in response to *Escherichia coli* infection compared to control cells due to reduced caspase-1 activation and IL-1β maturation (33). Nevertheless, there were no changes in the expression of NLRP3, apoptosis-associated speck-like protein (ASC), and pro-IL-1β mRNA suggesting that not the priming step but the inflammasome activation requires the presence of NLRC5. Using various infection models, it was found that NLRC5 is required for inflammasome activation by bacterial PAMPs and crystals, but not pore-forming toxins (33). Furthermore, co-immunoprecipitation studies showed that ectopically expressed NLRC5 exclusively associated with NLRP3 through the NLRC5-NBD domain (33). Albeit this is an interesting finding, we would like to note that, according to the experience of others and the authors (34), NBD domains of NLRs are prone to interact with each other resulting in eventual unphysiological complexes.

In consistence with these results, Yao and co-workers reported that reduced caspase-1 activation and IL-1β production was detected in BMDMs derived from their *Nlrc5^−/−^* mice upon stimulation with different NLRP3 inflammasome agonists such as monosodium urate crystals, the adjuvant alum, and LPS plus ATP (11). *In vivo* studies showed that NLRC5 contributed to *L. monocytogenes*-induced IL-1β secretion, and decreased neutrophil recruitment and bacterial clearance in the spleens and livers of infected *Nlrc5^−/−^* mice (11). However, no changes in pro-IL-1β mRNA were observed in this study, suggesting that NLRC5 might directly regulate the activation of the NLRP3 inflammasome.

In a model system, human rhinovirus (HRV)-induced IL-1β expression and caspase-1 activation were monitored in human primary epithelial cells, targeted with NLRC5-specific shRNA. Using Förster resonance energy transfer, the authors showed that following HRV infection, NLRP3 was associated with NLRC5 in primary bronchial cells. Furthermore, transfection of the viral 2B protein, a pore-forming toxin from the virus that enhances Ca^2+^ release from the stores, induced translocation of NLRC5 and NLRP3 from the cytoplasm to the Golgi apparatus to cooperatively sense intracellular Ca^2+^ fluxes and trigger IL-1β secretion (35).

Although these independent reports suggest that NLRC5 may affect NLRP3 inflammasome pathways in certain settings (Figure 2B), it should however be noted that most evidence that supports this function was obtained using reconstituted systems *in vitro*. These are prone to artifacts, and thus, more physiological data from endogenous NLRC5 should be used to establish a working model for an eventual function of NLRC5 in the regulation of inflammasomes.

## Type I Interferon Responses

Type I interferon responses are pivotal to control viral pathogens and contribute to immune defense in multiple pathways (36). Intriguingly, the first publication on NLRC5 by Kuenzle reported that NLRC5 is involved in the regulation of type I interferon responses (15). The authors show that human NLRC5 contains an IFNγ sensitive promoter and that NLRC5 mRNA is induced in several human cell lines and primary dermal fibroblasts by IFNγ treatment or cytomegalovirus (CMV) infection. Their work further revealed that NLRC5 might be able to target ISRE and gamma-activated sequence (GAS) promotor elements, although this finding was not confirmed by others (16). Finally, siRNA-mediated knockdown of NLRC5 led to reduced type I interferon responses toward CMV infection in HeLaS3 cells and primary human dermal fibroblasts (15). Similar results on the induction of NLRC5 expression and function were reported by Neerincx et al. using Sendai virus infection and poly(I:C) stimulation of human cell lines and primary human dermal fibroblasts (16).

Virus-induced induction of NLRC5 mRNA by respiratory syncytial virus and influenza A virus was subsequently shown to be dependent on the antiviral PRR retinoic acid-inducible gene I (RIG-I) (37, 38). This is mediated by an autocrine loop involving the signal transducer and activator of transcription 1 (STAT1) interferon pathway, elegantly shown by genetic depletion of STAT1 and IFNAR1 in mice (10, 29).

*In vitro* infection studies using human airway epithelial cells suggest a biological role of NLRC5 in antiviral immunity toward influenza A virus (PR8). Influenza virus replication was shown to be impaired by NLRC5 expression in human airway epithelial cells and type I interferon responses of a NS1 deletion strain were found to be partly dependent on NLRC5 in a RIG-I-dependent manner (38). Accordingly, overexpression of the NLRC5 homolog in zebrafish was shown to repress spring viremia of carp virus replication in fish cells and embryos (39). Several reports indicate that this function is mediated by physical interaction of NLRC5 with RIG-I (14, 38) and melanoma differentiation-associated protein 5 (MDA5) (14) and the signaling kinase TANK-binding kinase 1 (TBK1) (27). Accordingly, several groups reported that overexpression of NLRC5 in human embryonic kidney cells (HEK293T) results in reduced IFNγ-gene reporter activation upon triggering of the IFN pathway by TBK1 overexpression (14, 16, 27). Still, caution needs to be taken when interpreting the effects of NLR protein overexpression in gene reporter assays as these might lead to titration of co-actors and are prone to posttranslational effects, resulting in non-physiological effects (40). Knockdown and genetic ablation studies are more suited to avoid such shortcomings, although harbor pitfalls on their own. Surprisingly, the effect of siRNA-mediated knockdown of NLRC5 is opposing in reports from different groups (14–16). Consistent with the gene reporter assays, Cui et al. found that NLRC5 knockdown resulted in higher IFNβ responses upon poly(I:C) and vesicular stomatitis virus (VSV) treatment of human and murine monocytes (14). In a follow-up study, the same authors showed that NLRC5-deficient MEFs also have higher type I interferon responses upon VSV infection and confirmed *in vivo* that *Nlrc5^−/−^* mice have lower VSV titers and higher type I interferons (29). It should be noted that the mice used for this study were generated by targeting exon 8 of NLRC5, leaving the possibility that these animals still expressed a NLRC5 truncation protein without LRRs (29). This might be of a concern, as a NLRC5 construct lacking the LRRs is still able to repress influenza virus replication and responses (38). Indeed, an independent *Nlrc5^−/−^* mouse line, in which exon 4 was targeted, did not reveal any effect of NLRC5 on type I interferon responses, although this work was restricted to particular cell types, mainly GM-CSF-induced bone marrow dendritic cells (27).

Taken together, the *in vitro* data strongly suggest that NLRC5 can contribute to antiviral type I interferon responses in the cytosol *via* interaction with Rig-I, MDA5, and TBK1 (14, 27, 38) and possibly by direct targeting of ISRE and interferon-GAS promotor elements (15) (Figure 2C). The discrepancies between positive and negative effects and lack of contribution of NLRC5 to interferon responses in certain settings await careful assessment, leaving the biological role of NLRC5 in type I interferon responses elusive at present. Independent replication of these studies and controlled back-to-back comparison of the effect of NLRC5 depletion in different cell types and species will help to reveal the potential role of NLRC5 in type I interferon responses in the future.

## Malignant Transformation and Cell Cycle

Very recently, several reports from one lab showed that NLRC5 has a role in liver pathologies in hepatic fibrosis. NLRC5 is highly expressed in fibrotic liver tissue in mice and decreased expression was observed during resolution (41). Accordingly, NLRC5 mRNA expression is high in primary hepatocellular carcinoma (HCC) tissue and in cell lines such as HepG2, SMMC-7721, and BEL-7402 (42). In LX-2 hepatic stellate cells, TNF was shown to induce the expression of NLRC5 and NLRC5 can positively affect the expression of NF-κB target cytokines in these cells, likely involving Smad3 activation (43). In human keloid, a fibrotic tumor, characterized by extensive ECM deposition and hyperproliferation of fibroblasts in the skin, NLRC5 is also highly expressed. It was shown that in this tumor entity, NLRC5 knockdown suppressed cell proliferation and ECM deposition, involving transforming growth factor beta 1 (TGF-β1) and Smad2/3 (44). This suggests that NLRC5 might be able to interfere with Smad signaling in different malignant transformation processes.

Knockdown of NLRC5 in HepG2, SMMC-7721, and BEL-7402 cells resulted in somewhat lower cell proliferation rates and silencing of NLRC5 lead to G1 cell cycle arrest (42). Another study confirmed a correlation of NLRC5 expression and cell proliferation in HCC and suggested that NLRC5 modulates cell proliferation and the expression of VEGF-A in an AKT-dependent manner (45). There are also data available that link NLRC5 expression to cell migration and invasion, the most important features of malignant cell behavior. The authors show that NLRC5-induced activation of Wnt/catenin signaling pathway and subsequent expression of the Wnt target genes c-myc, cyclinD1, and MMP-7 might be causal for these effects (42), although these results await independent confirmation and validation *in vivo*.

To conclude, these reports suggest that NLRC5 might contribute to malignant transformation in multiple ways and in a tissue-dependent manner, likely by directly interfering with cell proliferation pathways (Figure 2D). Surprisingly, these results are opposing the established negative correlation of NLRC5 expression and tumor immunogenicity mediated by MHC class I-dependent antigen presentation and CTL-mediated restriction of malignant cells [reviewed in Ref. (46)]. Further research will provide more detailed analysis in different tumor entities and *in vivo* relevance to establish an eventual role and molecular function of NLRC5 in cell proliferation and cell signaling pathways.

## Conclusion

The function of many mammalian NLR proteins still is enigmatic. The identification of NLRC5 as the major MHC class I regulator added a lot to our understanding of the principles of immunology. However, many open questions regarding the biological function of this molecule remain to be answered. Both CIITA and NLRC5 are complex, high molecular weight proteins composed of highly structured domains that presumably require high energy consumption for folding and synthesis. In an evolutionary perspective, it is hard to imagine how such complex proteins remained conserved to function “only” as transcriptional activators. The involvement of many mammalian NLR proteins and their counterparts in autonomous immunity in plants also suggest additional functions. Notably, in plants, some NLR proteins function as sensors for pathogen invasion and mediate triggering of transcriptional activation in ways that are very similar to the function of CIITA and NLRC5 (2). Currently, it is unclear if NLRC5, and eventually CIITA, might function as PRRs or might have lost such functionality. For CIITA, no MHC II-independent function has been reported so far. The accumulating evidence discussed above, however, argues that NLRC5 at least takes part in the fine regulation of innate immune pathways. Structural information on NLRC5 and CIITA would help to get further insights into these functions. Unfortunately, NLR proteins are intrinsically difficult to express, likely a reason why most NLRs, including NLRC5, are expressed endogenously at very low levels.

In contrast to CIITA and the other NLRs, NLRC5 contains an unusual N-terminal domain, which shows some similarity to CARD domains (16, 25). This domain has intrinsic transcription activity and can transfer MHC II promotor activation when fused to CIITA (22). Future work revealing proteins that interact with this domain might provide insights into the function of this domain.

Given the well-established function of NLRC5 in MHC class I gene regulation, we also should be open minded to consider that changes in the expression of MHC I genes might be causal for, or at least contribute to the above discussed effects of NLRC5 on inflammatory responses. As it was shown that constitutive MHC I molecules can negatively affect the outcome of TLR stimulation in myeloid cells (47), it is conceivable that classical and eventually also the non-classical MHC molecules shape the outcome if innate immune responses more importantly as previously recognized.

At this time, it is very hard to draw a definite conclusion on the role of NLRC5 in innate immune responses. In particular, the mild phenotypes call for very careful and sophisticated analysis, which unfortunately often is not provided in the cited studies. It also should be reminded that NLRC5 is highest expressed in T cells in both human and mice (7, 10, 13, 15, 16), although nearly all cited work discussed above was conducted in myeloid and epithelial cells. Work in tissue and cells with higher basal expression of NLRC5 might help to yield more robust and physiological relevant data. Moreover, uncertainties about the genetic background of the different mouse strains used, off-target effects of siRNA and shRNA, and specificity of the used antibodies remain to be solved. Given that the NLRC5 field is only emerging, we are confident that applying improved technologies, such as CRISPR-Cas9, and progress in the recombinant expression of NLR proteins will lead to a better understanding of the biology of NLRC5 in the near future.

## Author Contributions

TK and SB conceived the manuscript, wrote a draft, and edited the text. FH added scientific discussions and proof-read and edited the manuscript. EK generated the figure and added helpful comments and scientific discussion.

## Conflict of Interest Statement

The authors declare that the research was conducted in the absence of any commercial or financial relationships that could be construed as a potential conflict of interest.
